# Investigation of Multilayer Edible Probiotic Coatings During Refrigerated Storage of Strawberries: A Focus on *Lacticaseibacillus casei* Shirota and *Lacticaseibacillus rhamnosus* GG †

**DOI:** 10.3390/microorganisms14102279

**Published:** 2026-10-07

**Authors:** Rozi Perndoj, Polyxeni Chryssanthou, Miltiadis Christopoulos, Anthoula Argyri, Chrysoula Tassou, Nikolaos Chorianopoulos, Anastasia E. Kapetanakou

**Affiliations:** 1 Institute of Technology of Agricultural Products, Hellenic Agricultural Organization-DIMITRA, S. Venizelou 1, 14123 Athens, Greece; 2 Laboratory of Microbiology and Biotechnology of Foods, Department of Food Science and Human Nutrition, School of Food and Nutritional Sciences, Agricultural University of Athens, Iera Odos 75, 11855 Athens, Greece

**Keywords:** strawberries, multilayer coating, active packaging, probiotics, *Lacticaseibacillus casei* Shirota, *Lacticaseibacillus rhamnosus* GG, shelf-life

## Abstract

The layer-by-layer (LBL) coating technique is an emerging alternative to conventional dipping for active packaging, improving coating adhesion to the hydrophilic surface of fruits. This study assessed the effects of probiotic multilayer coatings on microbial quality, physicochemical properties, and sensory attributes of strawberries stored in macro-perforated PET trays at 4 °C for 14 days. Strawberries were coated using a five-step LBL dipping process with alternating layers of 1% CaCl_2_ and 1% sodium alginate (SA). Strawberries coated with an LBL probiotic coating containing either *Lacticaseibacillus casei* Shirota (EC-LC) or *Lacticaseibacillus rhamnosus* GG (EC-LR), uncoated strawberries, and strawberries multilayer-coated with SA without probiotics (EC) were studied. Both probiotic-enriched coatings similarly inhibited (*p* ≥ 0.05) weight loss, decay, and TSS accumulation, thereby delaying ripening, while EC-LC improved the firmness of strawberries. Moreover, EC-LC was the most effective treatment in minimizing changes in sugars and organic acid concentrations. All coated samples showed a smaller decline in the a* parameter (redness) than controls, while EC-LC reached the highest values of lightness during storage, indicating a glossier appearance of the product. Fungal growth was inhibited by ca. 2.0 log CFU/g in EC-LC and EC-LR samples, while the embedded probiotic cultures remained viable, with counts near 10^6^ CFU/g of fruit during storage at 4 °C. Finally, sensory evaluation showed that uncoated strawberries became unacceptable by day 9, whereas EC-LC fruits remained acceptable in terms of odor and color attributes until day 14. Overall, it seems that the studied probiotic multilayer coatings, especially EL-LC, may offer new perspectives for extending the shelf-life of strawberries, while also providing them with functional properties.

## 1. Introduction

Strawberries (*Fragaria × ananassa* Duch.; Rosaceae family) are among the most widely consumed berries worldwide due to their attractive sensory attributes, high nutritional value, and abundance of bioactive compounds such as anthocyanins, vitamin C, and phenolic acids [1,2,3]. However, strawberries are highly perishable fruits, primarily due to their fragile epidermis, rapid softening (e.g., disassembly of pectin and other cell wall components), soft texture, and elevated metabolic activity, which render them particularly prone to mechanical damage during post-harvest handling, even under refrigerated storage [4,5]. In addition, the high respiration rate (20–40 mg CO_2_ kg^−1^ h^−1^ at 5 °C) and moisture content of strawberries accelerate quality deterioration and promote microbial proliferation (e.g., by fungi such as *Botrytis cinerea*), crucially affecting their shelf-life during storage and distribution [6,7,8]. In fact, according to the Food and Agriculture Organization [9], post-harvest losses of horticultural produce can be high, with ca. one-third never reaching human consumption, contributing significantly to food waste and economic losses, as well as posing a major challenge for the horticultural industry. Among the most common post-harvest methods to mitigate quality deterioration and extend strawberries’ shelf-life are rapid pre-storage cooling or hydrocooling, modified atmosphere packaging (MAP) (0–10% O_2_; 0–20% CO_2_), moderate hot air treatment, irradiation, and application of fungicides [6,8,10,11]. Despite the effectiveness of the aforementioned approaches, they present several limitations, including difficulties and high costs associated with temperature control, concerns about fungicide residues, the emergence of resistant pathogens, the formation of off-flavor compounds under anaerobic conditions, and undesirable color changes. Given the above, there is a growing demand for alternative, sustainable, and consumer-friendly post-harvest preservation interventions to maintain strawberry quality and extend their shelf-life.

In this context, active packaging technologies have gained increasing attention as innovative solutions for extending the shelf-life of fruits. Active packaging systems are designed to interact with the food or its surrounding environment to inhibit microbial growth, reduce oxidative reactions, and delay deterioration, thereby extending the product’s shelf-life and ensuring its safety [12]. Edible coatings are considered a promising form of active packaging, as they can act as a semi-permeable barrier to gases and moisture, preserving fruit quality, and/or as a carrier of active compounds such as antimicrobials or antioxidants, thereby enhancing product safety [13]. Moreover, edible coatings may contribute to reducing synthetic packaging waste due to their biodegradable nature [13,14]. Edible coatings formulated from various biopolymers, e.g., polysaccharides, proteins, and lipids, have been widely investigated for strawberries, demonstrating varying degrees of effectiveness in quality indices, e.g., in reducing weight loss, delaying softening, and inhibiting microbial growth [15,16,17]. However, conventional coating techniques for edible biopolymers, such as single-step dipping or spraying, often result in limited adhesion and non-uniform coverage, particularly on the hydrophilic, uneven surfaces of fruits like strawberries [18]. To overcome these limitations, the layer-by-layer (LBL) coating technique has emerged as a promising alternative, particularly for sodium alginate-based coatings [19]. This method involves the assembly of several sequential thin layers of oppositely charged polyelectrolytes on a charged substrate (e.g., the surface of strawberries), resulting in the formation of a stable, uniform, and adherent multilayer coating, in which the individual layers are chemically bonded through electrostatic interactions [20,21,22].

Probiotics, according to the Joint FAO/WHO Expert Consultation (2001) [23] definition, are “live microorganisms which, when administered in adequate amounts, confer a health benefit on the host”. Recently, edible coatings have attracted interest in incorporating probiotics, which offer bioprotective effects and potential health benefits such as improved gut microbiota and stronger immunity [24]. In fact, previous studies have shown that probiotic bacteria immobilized in edible coatings may remain viable above functional thresholds (10^6^–10^7^ CFU/g of food) during refrigerated storage, thus inhibiting spoilage microorganisms, slowing physiological deterioration, and preserving key quality attributes, e.g., weight, texture, and color of fresh produce [24,25,26]. Additionally, it has been proven that probiotic coatings (i.e., mucilage-based coating with *Lacticaseibacillus rhamnosus* GG or xanthan gum coatings with *Lactiplantibacillus plantarum* 75 and *Bifidobacterium longum*) may exhibit enhanced mechanical and barrier properties (e.g., reduced gas and moisture transfer), while creating localized environments that delay deterioration and oxidative damage [27,28]. However, the majority of studies are focused on probiotic-incorporated monolayer coatings [24,26,29], while limited information is available on the combined application of probiotic-loaded edible coatings using the advanced LBL technique for strawberries, particularly regarding their post-harvest shelf-life [30]. Furthermore, the potential synergistic benefits of integrating alginate-based matrices, probiotic functionality, and LBL deposition of the coating remain insufficiently studied and understood.

Considering the above, the current study aimed to assess the impact of sodium alginate-based coatings supplemented with either *Lacticaseibacillus casei* Shirota or *Lacticaseibacillus rhamnosus* GG, when applied using the LBL coating technique, on the microbial quality, physicochemical properties, and sensory attributes of strawberries during refrigerated storage.

## 2. Materials and Methods

### 2.1. Strawberry Selection, Preparation, and Handling

Commercial packages (500 g) of strawberries (*Fragaria × ananassa*; var. Victory) were purchased from a local supermarket (Athens, Greece) on the day of the experiment and immediately transported to the laboratory. To minimize sample variation, only fruits free from bruises, wounds, or other disorders were selected. Moreover, strawberries of similar size, color, and maturity were used. Subsequently, strawberries were washed by immersion in sterile distilled water and placed in a laminar flow cabinet to allow excess moisture to evaporate [31].

### 2.2. Probiotic Strains and Inoculum Preparation

The probiotic strains *Lcb. casei* Shirota (ACA-DC 6002) and *Lcb. rhamnosus* GG (ATCC 53103) were used throughout the study. Both species belong to the culture collection of the Institute of Technology of Agricultural Products, Hellenic Agricultural Organization-DIMITRA, and were preserved in De Man, Rogosa, and Sharpe broth (MRSB; Neogen^®^ Culture Media, Lansing, MI, USA) supplemented with 30% glycerol at −30 °C.

Before use, each frozen culture was activated by transferring 100 μL aliquots into 10 mL MRSB, followed by incubation at 30 °C for 24 h. Subsequently, aliquots of 1 mL from each overnight culture were transferred to 100 mL of fresh MRSB for a 24 h incubation at 30 °C. Afterwards, the individual species were collected by centrifugation at 6000× *g* for 10 min at 4 °C using a Heraeus Megafuge^TM^ 16R (Thermo Fisher Scientific, Tokyo, Japan). The harvested cells were washed twice with sterile ¼-strength Ringer’s solution (Neogen^®^ Culture Media, Lansing, MI, USA), and subsequently resuspended in 10 mL of ¼-strength Ringer’s solution to achieve a cell density of ca. 10^10^ CFU/mL. The population per species was determined by plating aliquots of 1 mL from the appropriate serial dilution onto double-layer De Man, Rogosa, and Sharpe agar (MRS; Neogen^®^ Culture Media, Lansing, MI, USA). The plates were then incubated at 30 °C for 48–72 h [32].

### 2.3. Preparation of Coating Solutions Incorporated With or Without the Probiotic Cultures of Lcb. casei Shirota or Lcb. rhamnosus GG

Sodium alginate (SA) solution was prepared following the protocol of Kapetanakou et al. (2019) [33]. Specifically, a 1% *w*/*v* SA solution was aseptically prepared by gradually dissolving 1 g of sterile SA powder (overnight UV-treated) (PanReac AppliChem ITW Reagents, Barcelona, Spain) in 100 mL sterile distilled water under constant stirring (MS 20A, WITEG Labortechnik GmbH, Wertheim, Germany) at 60 °C. After complete dissolution, sterile glycerol (1.0% *v*/*v*) was added to the SA solutions as a plasticizer to improve film flexibility, reduce brittleness, and modify coating moisture and oxygen permeability [34]. To prepare the probiotic coating solutions, 7 mL aliquots of freshly prepared cell suspensions of each strain (Section 2.2) were aseptically added to 700 mL of a 1% *w*/*v* SA solution and homogenized under constant stirring for ca. 20 min to obtain a final concentration of ca. 10^8^ CFU/mL of SA solution. The aforementioned probiotic population in the SA solution was enumerated by plating 1 mL of the appropriate serial dilution on double-layer MRS agar. The Petri dishes were incubated at 30 °C for 48–72 h. The probiotic coating solutions were prepared immediately after cell activation and harvesting (Section 2.2) to ensure maximum cell viability.

### 2.4. LBL Coating Technique and Refrigerated Storage of Strawberries

**Figure 1:**
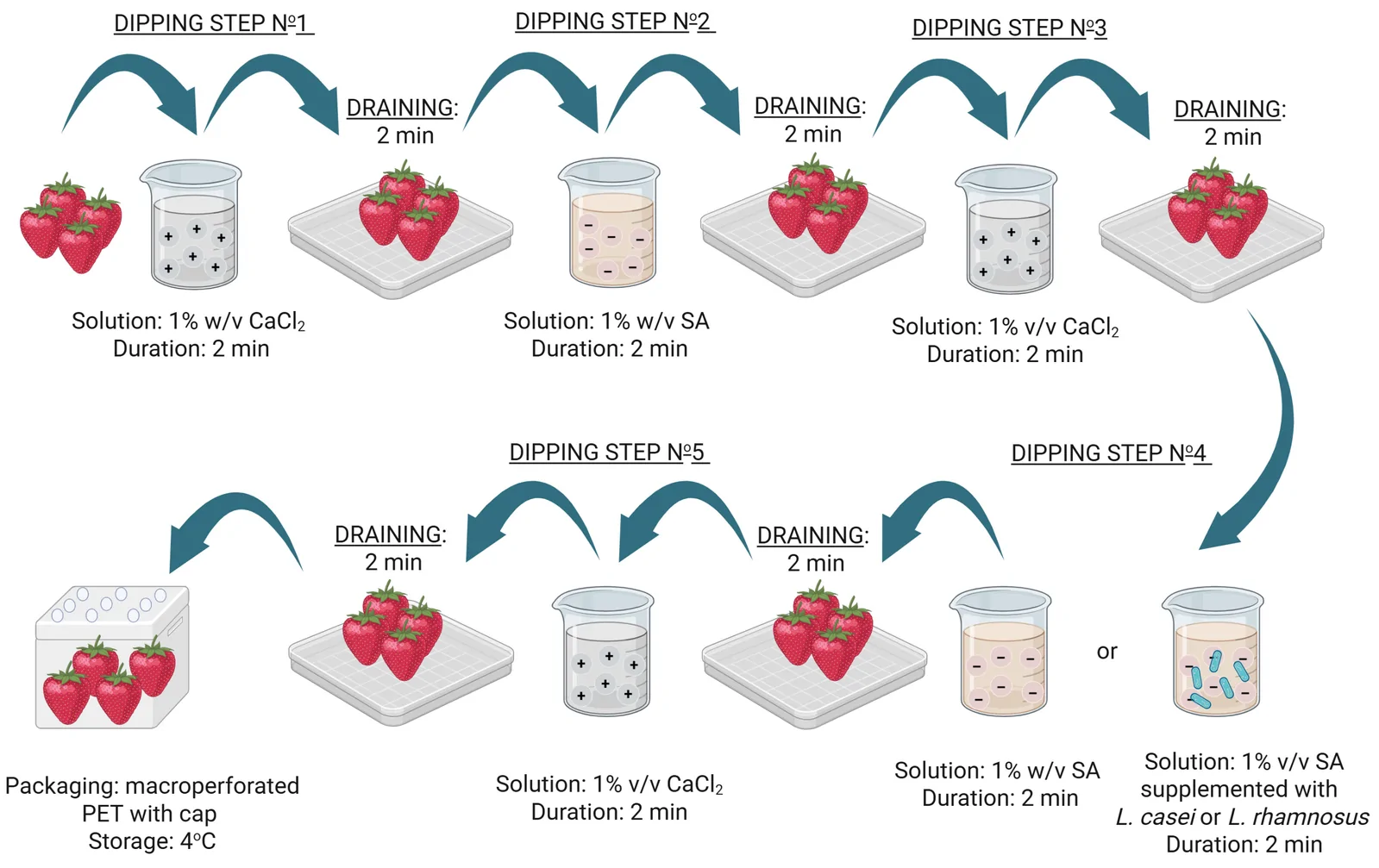
Illustration of the sequential dipping steps of the LBL coating technique in strawberries with SA supplemented with *Lbc. casei* Shirota or *Lbc. rhamnosus* GG or without probiotic cultures and packaged in food-contact macro-perforated PET containers during storage at 4 °C [35] (Created in BioRender. Kapetanakou, A.E. (2025). https://app.biorender.com/illustrations/6889c52e4dc1a67720e0c282?slideId=8602636a-88d6-415f-b684-8e%E2%80%A6).

Strawberries were coated using the LBL coating technique, which involves 5 sequential dipping steps, each lasting 2 min, following a modified protocol of Sipahi et al. (2013) [35]. Specifically, the dipping sequence was (Figure 1): 1% *w*/*v* calcium chloride (CaCl_2_) (PanReac AppliChem ITW Reagents, Barcelona, Spain) (Dipping Step No. 1), 1% *w*/*v* SA (Dipping Step No. 2), 1% *w*/*v* CaCl_2_ (Dipping Step No. 3), 1% *w*/*v* SA supplemented with *Lcb. casei* Shirota or *Lcb. rhamnosus* GG (ca. 8.0 log CFU/mL) (Dipping Step No. 4), and 1% *w*/*v* CaCl_2_ (Dipping Step No. 5). Following each dipping step, the strawberries were allowed to drain for 2 min to eliminate any excess coating solution. Thus, the 4 experimental assays studied were strawberries (i) without coating, which served as controls, (ii) multilayer-coated with the SA solution without containing probiotic cultures (coded as EC), and (iii) multilayer-coated with the SA solution containing either *Lcb. casei* Shirota (EC-LC) or *Lcb. rhamnosus* GG (EC-LR). The initial concentration of viable probiotic population in SA-coated strawberries was set at ca. 6.0 log CFU/g (enumerated in double-layer MRS agar after incubation at 30 °C for 48–72 h) [24,25,26]. This decision was based on the assumption that a typical minimum serving of strawberries is 100 g. Based on that, the total probiotic intake would be ca. 8.0 log CFU, a sufficient population to preserve their functional benefits [36]. Four strawberries (ca. 100 ± 10 g) per experimental assay were placed inside a food-contact square PET clamshell container (500 mL) with a macro-perforated cap. The samples were maintained at 4 °C for 14 days in high-precision incubation chambers with a temperature tolerance of ±0.5 °C (MIR-154 Cooled Incubator, PHCbi, Tokyo, Japan). PET containers were sterilized via exposure to ultraviolet light for 20 min before use. Three samples were prepared for each treatment, and the storage study was conducted in duplicate experiments (*n* = 6).

### 2.5. Physicochemical Analysis

#### 2.5.1. Weight Loss

Each package was weighed on day 0 and at each sampling point using an electronic precision balance (PFB 2000-2, KERN & SOHN GmbH, Balingen, Germany). Weight loss was calculated according to the following equation [31]:

(1) $$\mathrm{Weight}\;\mathrm{Loss}\;(\%)=\frac{W_{o}-W_{s}}{W_{o}}\times 100$$

where *W_o_* is the weight (g) on day 0, and *W_s_* is the measured weight (g) on each sampling day.

#### 2.5.2. Visible Decay

At each sampling interval, the individual packages were opened, and the strawberries were visually examined for macroscopic signs of deterioration, i.e., discoloration, shrinkage, tissue softening, or fungal growth. Visible decay was calculated according to the following equation [31]:

(2) $$\mathrm{Visible}\;\mathrm{decay}\;(\%)=\frac{\mathrm{Number}\;\mathrm{of}\;\mathrm{decayed}\;\mathrm{strawberries}}{\mathrm{Total}\;\mathrm{number}\;\mathrm{of}\;\mathrm{strawberries}}\times 100$$

#### 2.5.3. Titratable Acidity, Total Soluble Solids Content, and Ripening Index

At each sampling point, 2–3 strawberries were randomly selected and homogenized using a household blender. Five grams of homogenized strawberries were weighed, placed in a 50 mL volumetric flask, and diluted with deionized water. The diluted strawberry homogenate was placed in an ultrasonic bath (IBX Instruments, Barcelona, Spain) for 5 min. Afterwards, the mixture was filtered through a 0.45 μm PVDF syringe filter (Membrane Solutions, Washington, DC, USA). Titratable acidity (TA) was expressed as % citric acid (g of citric acid in 100 mL of strawberry juice) following titration of 10 mL of the filtrate with 0.1 N NaOH and the addition of 2–3 drops of phenolphthalein (as an indicator) to a pH endpoint of 8.2 [26]. TA was calculated based on the following equation:

(3) $$\mathrm{TA}\;\left(\%\right)=\frac{V\;\times N\;\times K\;\times V_{0\;}}{m\;\times \;V_{1}}\;\times 100$$

where *V* is the volume of NaOH consumed (mL), *N* is the concentration of NaOH standard solution (0.1 mol/L), *K* is the equivalent weight of citric acid (0.064 g/mmol), *V*_0_ is the total solution volume, *m* is the mass of the homogenate (g), and *V*_1_ is the volume of filtrate used for titration.

Total soluble solids (TSS) were measured at room temperature using a handheld refractometer (Atago Co. Ltd., Tokyo, Japan). Following homogenization of strawberries from each treatment group, 2–3 drops of the resulting filtrate were applied to the prism surface for direct measurement, and the results were recorded in °Brix.

Finally, the ripening index (*RI*), a parameter commonly used to quantify the maturity of fruits, was calculated by the equation below [37]:

(4) $$\mathrm{RI}\;=\frac{\mathrm{TSS}}{\mathrm{TA}}$$

#### 2.5.4. Firmness

The firmness of strawberries was assessed following a modified version of the method described by Zhang et al. (2018) [38]. The maximum penetration force (F_max_) was determined using a texture analyzer (TA.HDplusC, Stable Micro Systems, Surrey, UK) equipped with a cylindrical probe having a diameter of 2 mm. For each measurement, the probe was inserted to a depth of 10 mm into each sample, and the crosshead speed of the texture analyzer was set at 10 mm/s. Firmness was reported as the maximum force (N) required for penetration. Measurements were taken in 8 randomly selected strawberries per treatment at each sampling point.

#### 2.5.5. Color

Skin color of strawberries was assessed using the CIE L*a*b* color system by measuring lightness (L*), redness (a*), and yellowness (b*) parameters obtained using a portable CR-300 series spectrocolorimeter (Konica Minolta Inc., Tokyo, Japan) [29]. In this system, L* represents lightness on a scale from 0 (black) to 100 (white), whereas positive and negative a* values correspond to red and green hues, respectively. Similarly, positive b* values indicate yellow coloration, while negative values represent blue coloration. Before analysis, the instrument was calibrated against a white reference standard (L* = 100). Ten readings were obtained using 8 randomly selected strawberries per treatment at each sampling point (*n* = 80).

#### 2.5.6. Microbiological Analysis and pH Measurement

At each sampling point, ca. 10 g of strawberry samples per treatment was aseptically mixed with 90 mL of sterile Ringer’s solution (Lab M, Lancashire, UK) in a sterile stomacher bag and homogenized in a stomacher (Interscience, Saint-Nom-la-Bretèche, France) for 60 s. Following homogenization, serial 10-fold dilutions in ¼-strength Ringer’s solution were prepared, and aliquots of 1 or 0.1 mL from the appropriate dilution were poured or spread onto selective and non-selective culture media, respectively. Specifically, Total Viable Counts (TVC) were enumerated on Plate Count Agar (PCA; BIOKAR Diagnostics, Allonne, France) after incubation at 30 °C for 48 h; presumptive lactic acid bacteria (LAB) on a double layer of MRS agar (pH 5.8) at 30 °C for 48–72 h; and yeasts and molds on Rose Bengal Chloramphenicol Agar (RBC; BIOKAR Diagnostics, Allonne, France) incubated at 25 °C for 96 h [39]. Results were expressed as log CFU/g of strawberries.

The pH values were recorded throughout storage using a digital pH meter HI2211 (Hanna Instruments Inc., Smithfield, RI, USA) by immersing the glass electrode in strawberry homogenate following microbiological analysis.

#### 2.5.7. Quantification of Organic Acids and Sugars via HPLC-UV/RI

Two grams of homogenized strawberries were weighed and added to a 20 mL volumetric flask with deionized water. The diluted strawberry homogenate was placed in an ultrasonic bath for 30 min, and each sample was filtered using a 0.45 μm PVDF syringe filter. The analysis was performed according to Hwang et al. (2019) [40] using an Agilent 1100 series High-Performance Liquid Chromatography system (Agilent Technologies, Inc., Santa Clara, CA, USA) equipped with a G1314A Variable Wavelength Detector and a Rheodyne HPLC manual injector, Model 7010. Separation was performed using a cation-exchange Repromer H column (9 µm; 300 × 7.8 mm; Dr. Maisch GmbH, Ammerbuch, Germany). The sample injection volume was 10 µL. Elution was carried out isocratically using a 1 mM H_2_SO_4_ solution as the mobile phase at a flow rate of 0.7 mL/min, and the column temperature was set to 50 °C. The processing and acquisition of chromatograms were carried out using Agilent ChemStation software (B.02.01.SR1 Revision, Agilent Technologies, Inc., Santa Clara, CA, USA). To quantify the concentrations of sugars and organic acids, calibration curves were generated over the ranges 0.01–0.1% (*w*/*v*) and 0.1–0.5% (*w*/*v*). Two samples from two independent storage experiments per experimental assay were analyzed (*n* = 4). Results were expressed as g/100 g of fruit.

#### 2.5.8. Sensory Evaluation

Sensory evaluation of strawberries was conducted on days 0, 5, 9, and 14 under artificial lighting and at room temperature. The evaluation panel consisted of ten members of the laboratory staff from the Institute of Technology of Agricultural Products, Hellenic Agricultural Organization-DIMITRA. The panel assessed strawberries from each packaging treatment for color (i.e., brightness, uniformity, and presence of browning or discoloration), odor (i.e., intensity and presence of off-odors), texture (i.e., firmness, juiciness, and surface integrity), and overall freshness, defined as the integrated visual perception of freshness, including color uniformity, juiciness, and surface integrity. Before sensory assessment, panelists received comprehensive instructions on the sensory attributes they would evaluate and signed the informed consent form. Samples were offered to the panelists on white plastic plates coded with 4 random numbers (blind evaluation). Each attribute was assessed on a 5-point hedonic scale from 1.0 (unacceptable—spoiled) to 3.0 (acceptable—fresh) with 0.5 intervals. Samples were considered acceptable when they received scores higher than 2, while scores below 2 indicated that samples were unacceptable to the testing panel. A score of 2.0 indicated that strawberries changed from typical fresh to not fresh; however, they were still considered acceptable.

### 2.6. Statistical Analysis

Statistical analysis was conducted using SPSS software, Version 16.0 (SPSS Inc., Chicago, IL, USA). Analysis of variance (ANOVA) was applied to assess the effects of treatment, storage time, and their interactions on the quality parameters, microbial counts, and sensory characteristics. Differences among means were evaluated using Tukey’s b multiple range test and *t*-tests at a 95% confidence level.

## 3. Results and Discussion

### 3.1. Weight Loss

**Figure 2:**
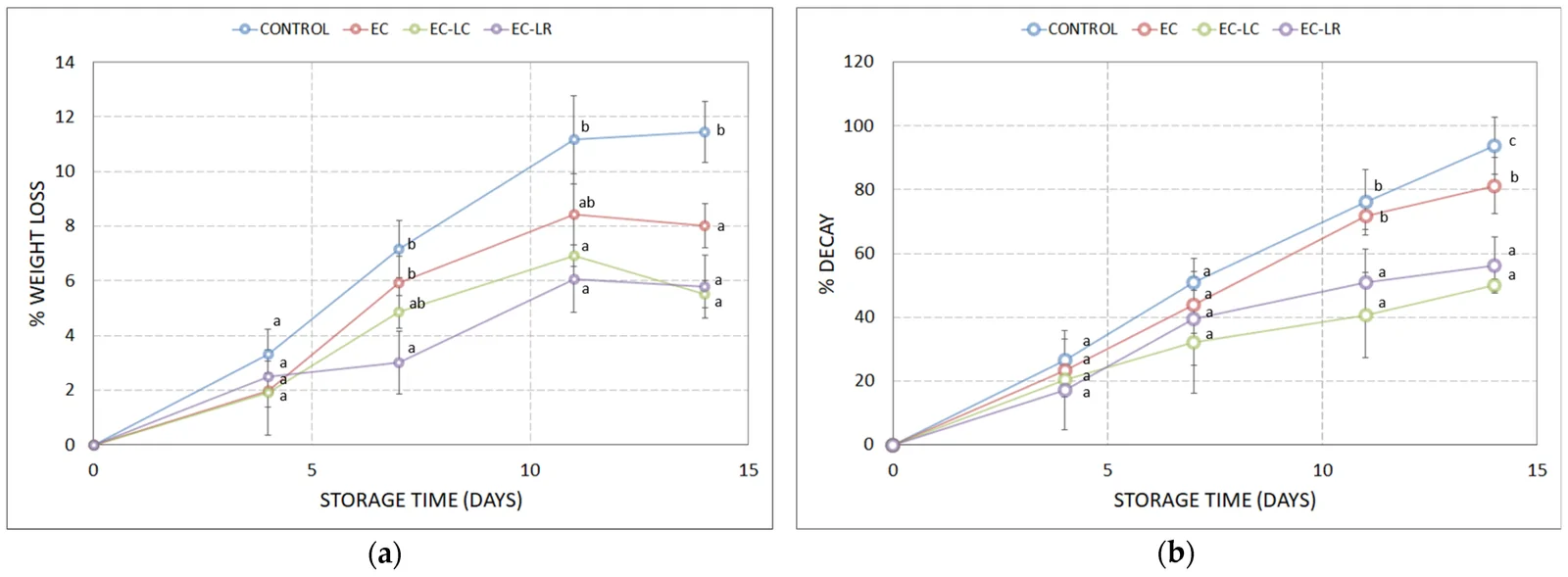
Variation in (**a**) % weight loss and (**b**) % visible decay of strawberries coated with multilayer SA coatings supplemented with either *Lbc. casei* Shirota (EC-LC) or *Lbc. rhamnosus* GG (EC-LR) or no probiotic cultures (EC), packaged in food-contact macro-perforated PET containers and stored at 4 °C. Uncoated strawberries served as controls. Different lowercase letters within the same sampling day indicate statistically significant differences among treatments (*p* < 0.05).

During storage, weight loss of strawberries progressively increased (*p* < 0.05) in all studied treatments; however, samples coated with SA multilayer coatings (either containing probiotics or not) showed significantly lower weight loss (*p* < 0.05) compared to controls throughout storage (Figure 2a). Weight loss in fruits mainly occurs due to: (i) breakdown of carbohydrates with release of CO_2_ (carbon loss) and water through respiration and (ii) the evaporation of water from the surface of fruit driven by vapor pressure gradients via transpiration [7]. In fact, both physiological processes are crucially accelerated after harvest. Specifically, on day 14, controls exhibited the greatest weight loss (*p* < 0.05), reaching 11.45 ± 1.13%. At the same time, SA-coated strawberries without probiotic cultures (EC samples) showed significantly (*p* < 0.05) lower weight loss, namely 8.03 ± 0.81% (Figure 2a), than controls. The aforementioned results indicate that SA coating in multiple layers can serve as an effective semi-permeable barrier, inhibiting water and gas migration and reducing the weight loss of strawberries. Similar to our results, Adhikari et al. (2023) observed weight loss of ca. 13.05% in strawberries multilayer-coated with chitosan–pectin (nine layers) compared to uncoated samples (44.52%) after 9-day storage at 25 °C [30]. On the other hand, Parreidt et al. (2019) showed that the application of SA monolayer coating promoted water loss in fresh-cut strawberries during storage at 10 °C [41]. Previous studies have also highlighted the effective moisture barrier properties of multilayer coatings of various biopolymers when applied solely (e.g., alginate/pectin, whey protein isolate/chitosan) during refrigerated storage in various fruits, e.g., fresh-cut cherimoya, pineapple, or watermelon, thus leading to a reduction in dehydration and minimizing fruits’ shriveling [35,42,43]. Moreover, the incorporation of probiotic cultures into the multilayer SA coating resulted in a significantly lower (*p* < 0.05) percentage of weight loss, namely EC-LC: 4.86 ± 0.59% and EC-LR: 3.01 ± 1.14% compared with the uncoated (7.16 ± 1.05%) and the EC samples (5.91 ± 1.01%), with this difference being evident from day 7 (Figure 2a). No significant differences (*p* ≥ 0.05) were recorded between the two probiotic cultures studied. Similar to our results, Wong et al. (2021) reported ca. 1.0% weight loss in fresh-cut apple slices bilayer-coated with carboxymethyl cellulose (CMC)/zein supplemented with *Lactobacillus plantarum* 299v after 7 days at 4 °C compared to uncoated samples (6.0 ± 2.2%) [44].

### 3.2. Visible Decay

The percentage of visible decay in strawberries increased (*p* < 0.05) during storage at 4 °C in all the assays studied; however, significant differences were recorded among the applied treatments (Figure 2b). Specifically, the % visible decay rate increased following the order control > EC > EC-LR = EC-LC during storage (Figure 2b). In fact, controls exhibited the highest visible decay rate, reaching 94 ± 9% on day 14 (with about ca. 50% of the samples spoiled by day 7), while strawberries coated with *Lbc. casei* or *Lbc. rhamnosus* showed considerably lower rates, namely 56 ± 9% (EC-LR) and 50 ± 2% (EC-LC), respectively (Figure 2b). However, no significant differences (*p* ≥ 0.05) were recorded between the two probiotic cultures studied. Regarding the EC samples, the visible decay recorded at the end of storage was significantly lower 81 ± 9% (*p* < 0.05) compared to controls (94 ± 9%), further highlighting the cumulative effect of incorporating both probiotic cultures into the multilayer alginate coating (Figure 2b). The recorded increased effectiveness in the % visible decay of the studied probiotic multilayer coating *versus* EC or controls is mainly attributed to the antimicrobial properties of probiotic cultures, which suppress the metabolic activity and growth of spoilage microbiota that commonly infect strawberries, such as fungi. The latter interpretation is supported by the significant inhibition (*p* < 0.05) of the enumerated fungi in EC-LC (3.0 ± 0.1 log CFU/g) and EC-LR (2.7 ± 0.1 log CFU/g) samples compared to controls and EC (4.3 ± 0.5 log CFU/g) samples after 14 days at 4 °C (Figure 5b). In agreement with our results, Khodaei and Hamidi-Esfahani (2019) showed that increasing the concentration of *L. plantarum* from 0 to 9.80 × 10^3^ CFU/mL in a CMC coating reduced the % visible decay of strawberries from 60% to 40% after 16 days of storage at 4 °C [29].

### 3.3. PH, Titratable Acidity, Soluble Solids Content, and Ripening Index

**Figure 3:**
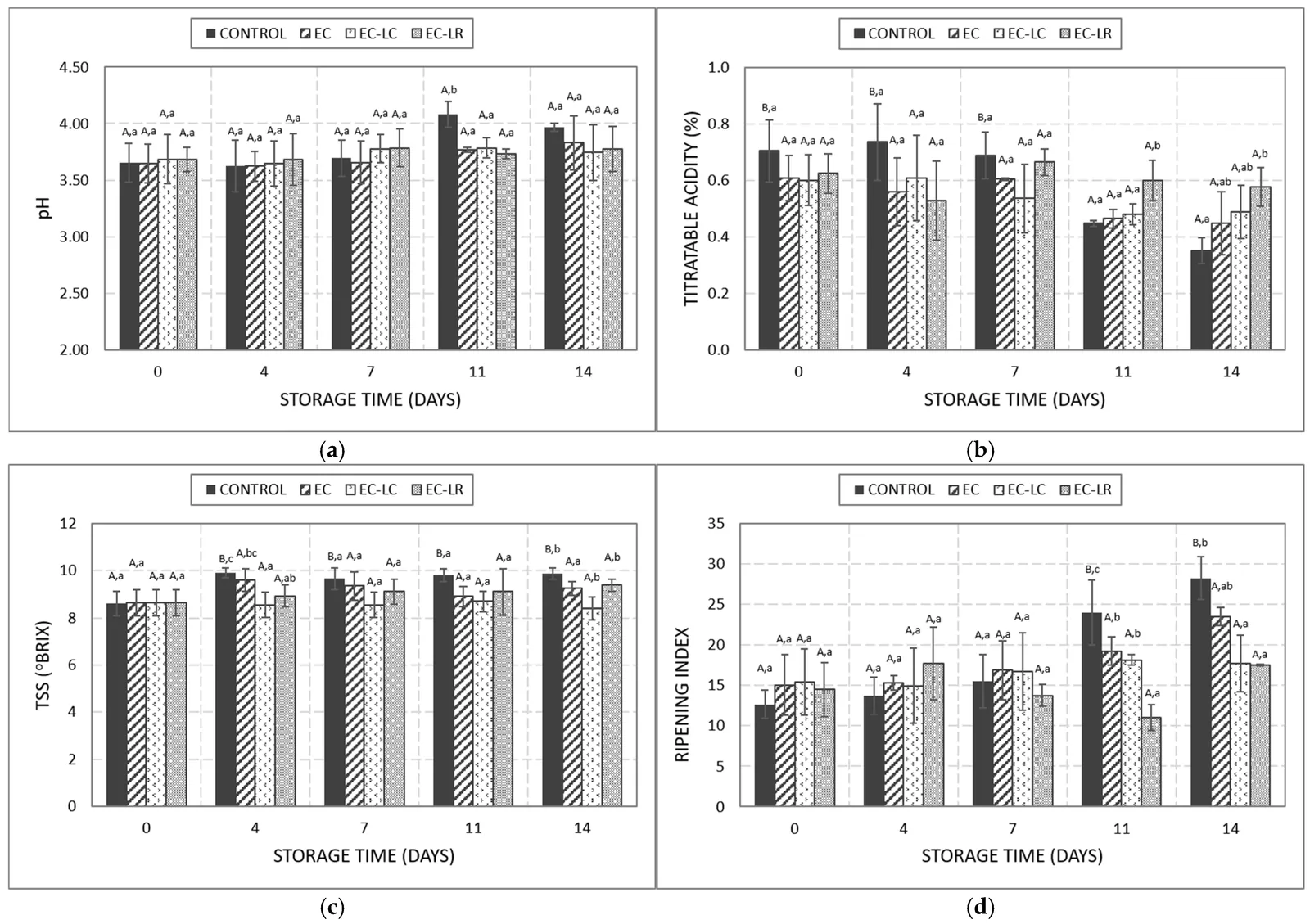
Variation in (**a**) pH, (**b**) TA (%), (**c**) TSS (°Brix), and (**d**) RI of strawberries coated with multilayer SA edible coatings supplemented with either *Lbc. casei* Shirota (EC-LC) or *Lbc. rhamnosus* GG (EC-LR) or no probiotic cultures (EC), packaged in food-contact macro-perforated PET containers and stored at 4 °C. Uncoated strawberries served as controls. Different uppercase letters within the same treatment indicate statistically significant differences among sampling days (*p* < 0.05). Different lowercase letters within the same sampling day indicate statistically significant differences among treatments (*p* < 0.05).

The effect of different treatments on pH, TA, TSS, and RI during storage of strawberries is shown in Figure 3. In controls, pH was slightly increased from 3.65 ± 0.17 on day 0 to 4.08 ± 0.11 (Figure 3a). The latter result agrees with previous researchers studying post-harvest shelf-life in various varieties of strawberries like Selva, and Driscoll [45,46], since the dominant organic acids like citric acid and/or malic acid are metabolized during post-harvest respiration of strawberries. This result is also supported by the decrease (*p* < 0.05) in citric and malic acids recorded in untreated strawberries (Table 2). In contrast, EC, EC-LC and EC-LR samples showed stable pH values (*p* ≥ 0.05) throughout the 14-day storage, ranging from 3.65 ± 0.17 to 3.83 ± 0.24, 3.69 ± 0.22 to 3.75 ± 0.25, and 3.68 ± 0.11 to 3.78 ± 0.20, respectively (Figure 3a). This effect is mainly attributed to the ability of SA-based coatings to function as a partial oxygen barrier, thereby reducing the respiration rate, and contributing to the stabilization of pH during storage [19]. Similar to our results, previous studies have shown that monolayer CMC coatings containing *L. plantarum* or gelatin coatings containing *L. rhamnosus* and inulin may contribute to greater pH stability in strawberries compared to uncoated samples or those coated solely with CMC or gelatin during storage at 4 °C [26,29]. A similar trend was observed in TA among the different treatments; namely, strawberries multilayer-coated with (EC-LC and EC-LR) or without probiotics (EC) exhibited greater stability (*p* ≥ 0.05) compared to controls throughout storage. Specifically, TA values for EC, EC-LC, and EC-LR samples ranged from 0.61 ± 0.08% (day 0) to 0.45 ± 0.11% (day 14), 0.60 ± 0.09% (day 0) to 0.49 ± 0.10% (day 14), and 0.62 ± 0.07% to 0.58 ± 0.07% (day 14), respectively (Figure 3b). Regarding the controls, TA remained stable (*p* ≥ 0.05) until mid-storage but then significantly declined (*p* < 0.05) from 0.70 ± 0.11% (day 7) to 0.35 ± 0.05% (day 14) (Figure 3b), a result that comes in agreement with the slight increase in pH (Figure 3a). At the same time, TSS in controls showed a significant increase (*p* < 0.05) even from day 4 (9.90 ± 0.20 °Brix) compared to day 0 (8.60 ± 0.52 °Brix) and remained stable (9.80–9.95 °Brix) until the end of storage (*p* ≥ 0.05) (Figure 3c). The latter result is well associated with the recorded weight loss (Figure 2a) in controls and the consequent sugar condensation. On the contrary, TSS levels for all coated samples, regardless of probiotic cultures, remained stable and similar to day 0 values (*p* ≥ 0.05) (Figure 3c). Contrary to our results, Khodaei and Hamidi-Esfahani (2019) reported that the TSS of strawberries was not influenced when different amounts of *L. plantarum* were incorporated into CMC monolayer edible coatings during storage at 4 °C for 15 days [29]. However, studies investigating LBL coatings (without probiotics) have shown significant inhibition of TSS accumulation in coated strawberries (i.e., chitosan and pectin) compared with uncoated samples [40]. Regarding the % RI, controls showed a significant increase (*p* < 0.05) from 13% to 33%, while at the same time, EC, EC-LC, and EC-LR samples revealed a restricted increase after 14 days, namely ca. 24, 15, and 15%, respectively (Figure 3d). Overall, it seems that EC-LC and EC-LR coatings significantly (*p* < 0.05) delayed ripening of strawberries and in similar percentages (*p* ≥ 0.05), as was also macroscopically evident (Figure 7).

### 3.4. Color

Table 1 presents the effects of the different treatments on strawberry color parameters during the 14-day storage. Controls exhibited no significant changes in lightness (*p* ≥ 0.05), with L* values ranging from 35.77 ± 0.13 on day 0 to 36.18 ± 0.16 on day 14. In contrast, all coated samples (EC, EC-LC, and EC-LR) showed a significant increase in L* values (*p* < 0.05) even after day 4, with EC-LC reaching the highest value on day 7 (41.57 ± 2.30), indicating a glossier appearance of the product. Although L* decreased slightly toward day 14, all treated samples consistently showed higher L* values than controls, suggesting that the SA coating contributed to preserving surface appearance. Concerning the a* parameter (redness), controls showed a significant decline (*p* < 0.05) from 35.91 ± 0.03 (day 0) to 30.83 ± 0.50 (day 14). In all coated samples, the initial a* values were lower (31.02 ± 0.08) than those of the controls (35.91 ± 0.03) (*p* < 0.05); however, they showed a smaller decline at the end of 14-day storage (28.72–29.35) compared to uncoated strawberries (Table 1). Regarding the b* parameter (yellowness), controls showed a significant decrease (*p* < 0.05) from 23.02 ± 0.42 (on day 0) to 21.98 ± 0.21 (on day 14), while all coated samples maintained lower and relatively stable b* values, ranging from 17.78 ± 1.07 to 19.85 ± 1.61, from 17.13 ± 1.79 to 18.86 ± 0.94 for EC-LR, and from 18.19 ± 0.02 to 20.48 ± 0.96 for EC-LC (Table 1). The above results indicate that coatings with or without probiotics were effective at preserving strawberry color, likely by reducing oxidative changes and delaying anthocyanin degradation. Overall, the application of the two probiotic cultures (EC-LC and EC-LR) affected color parameters similarly (*p* > 0.05) throughout storage at 4 °C. Similar were the results of Khodaei and Hamidi-Esfahani et al. (2019), who reported that strawberries coated with CMC supplemented with *L. plantarum* maintained stable L*, a*, and b* values during storage at 4 °C compared to controls [29]. Previous researchers in other fruits, such as fresh-cut kiwis, fresh-cut melons, and apple chips coated with probiotic-loaded konjac gum, xanthan gum, and alginate/gelatin coatings, respectively, have also reported stability in their color [27,47,48].

### 3.5. Firmness

**Figure 4:**
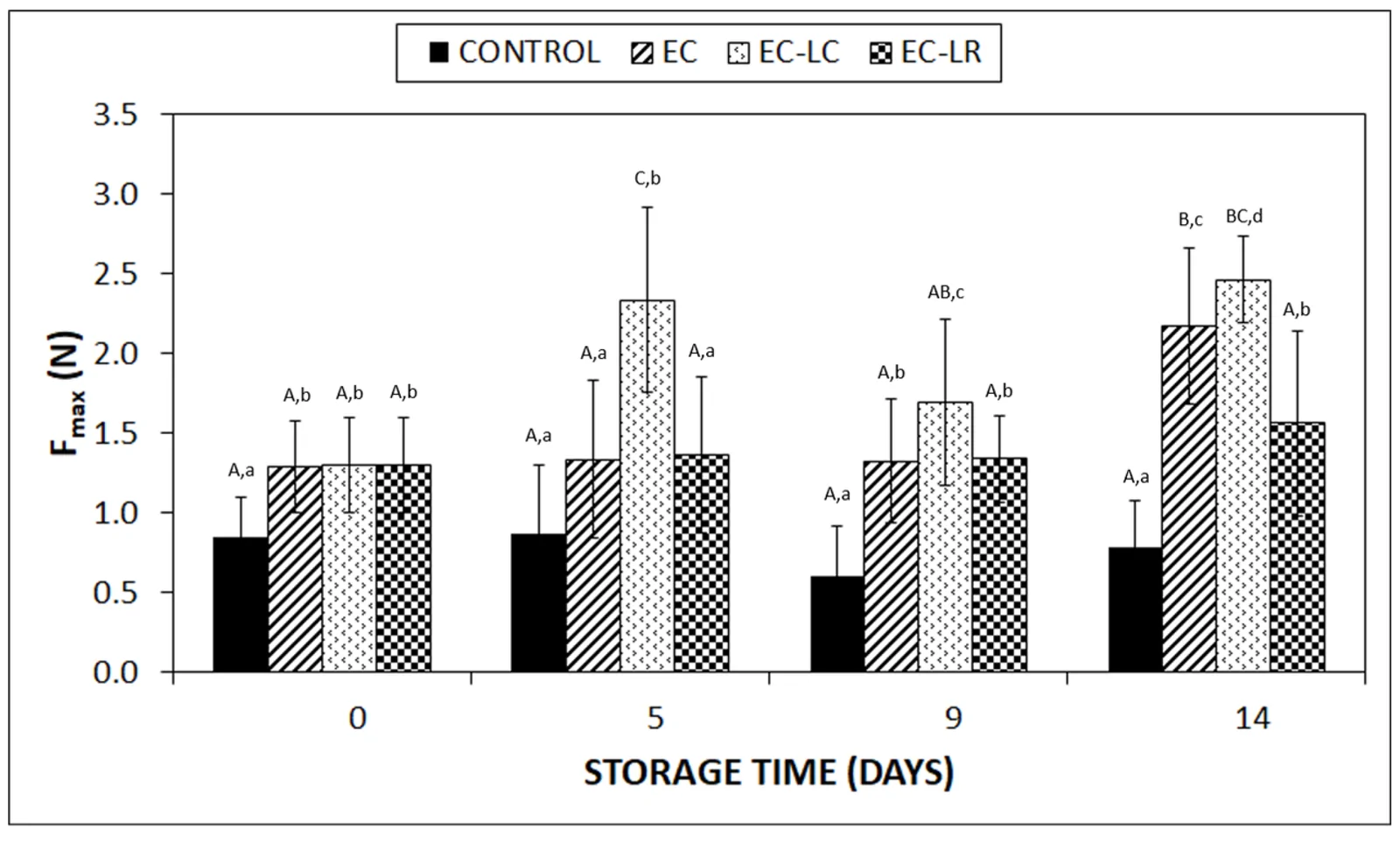
Variation in F_max_ (N) of strawberries coated with multilayer SA edible coatings supplemented with either *Lbc. casei* Shirota (EC-LC) or *Lbc. rhamnosus* GG (EC-LR) or no probiotic cultures (EC), packaged in food-contact macro-perforated PET containers and stored at 4 °C. Uncoated strawberries served as controls. Different uppercase letters within the same treatment indicate statistically significant differences among sampling days (*p* < 0.05). Different lowercase letters within the same sampling day indicate statistically significant differences among treatments (*p* < 0.05).

Figure 4 illustrates the variations in strawberry firmness observed over the 14-day storage period. On day 0, all coated samples, with or without probiotics, showed significantly higher firmness (*p* < 0.05) compared to controls, potentially due to the presence of SA multiple-layer coating. Niam et al. (2025) studied various formulations containing three biopolymers (alginate, guar gum, and pectin) and reported that higher alginate concentrations within the composite strengthen the gel network, supporting strawberry texture from day 0 [49]. During storage, controls exhibited the lowest recorded F_max_ values (*p* < 0.05), ranging from 0.6 to 0.9 N compared to coated samples, indicating greater tissue softening potentially due to higher % weight loss (Figure 2a) and cell wall degradation, characteristics which are considered typical when post-harvest deterioration takes place. Specifically, all coated samples showed a gradual increase in F_max_ starting from 1.3 ± 0.3 N on day 0 and reaching a range of 1.6–2.5 N on day 14, thus demonstrating their ability to delay textural degradation and enhance the structural integrity of strawberries (Figure 4). This increase may be related to an interaction between the coating matrix and the fruits’ surface and/or surface desiccation, forming a tougher outer coating layer. Similarly, Adhikari et al. (2023) observed an increase in firmness of multilayer-coated strawberries with chitosan–pectin (nine layers) (3.9 ± 0.2 N) after 9-day storage at 25 °C compared to uncoated samples (1.3 ± 0.2 N) [30]. Regarding the probiotic-enriched coatings, *Lbc. casei* Shirota (EC-LC) exhibited, even from day 5, the highest F_max_ values (2.3 ± 0.6 N), being ca. 2- to 3-fold higher than EC-LR (1.4 ± 0.5 N), EC (1.3 ± 0.5 N), and controls (0.9 ± 0.4 N). Previous studies have reported similar enhancement of firmness when SA probiotic-enriched coatings (i.e., *Lpb. plantarum* subsp. *plantarum*; *Lactobacillus rhamnosus*; *Bifidobacterium animalis subsp. lactis*) were applied not only to strawberries [24,26] but also to other fruits such as fresh-cut mangoes and apples [50,51]. The above findings clearly indicate that, among the applied probiotic treatments, the application of *Lcb. casei* Shirota into multilayer SA coating (EC-LC) on strawberries may enhance the mechanical resistance and help preserve their textural integrity.

### 3.6. Microbiological Analysis

**Figure 5:**
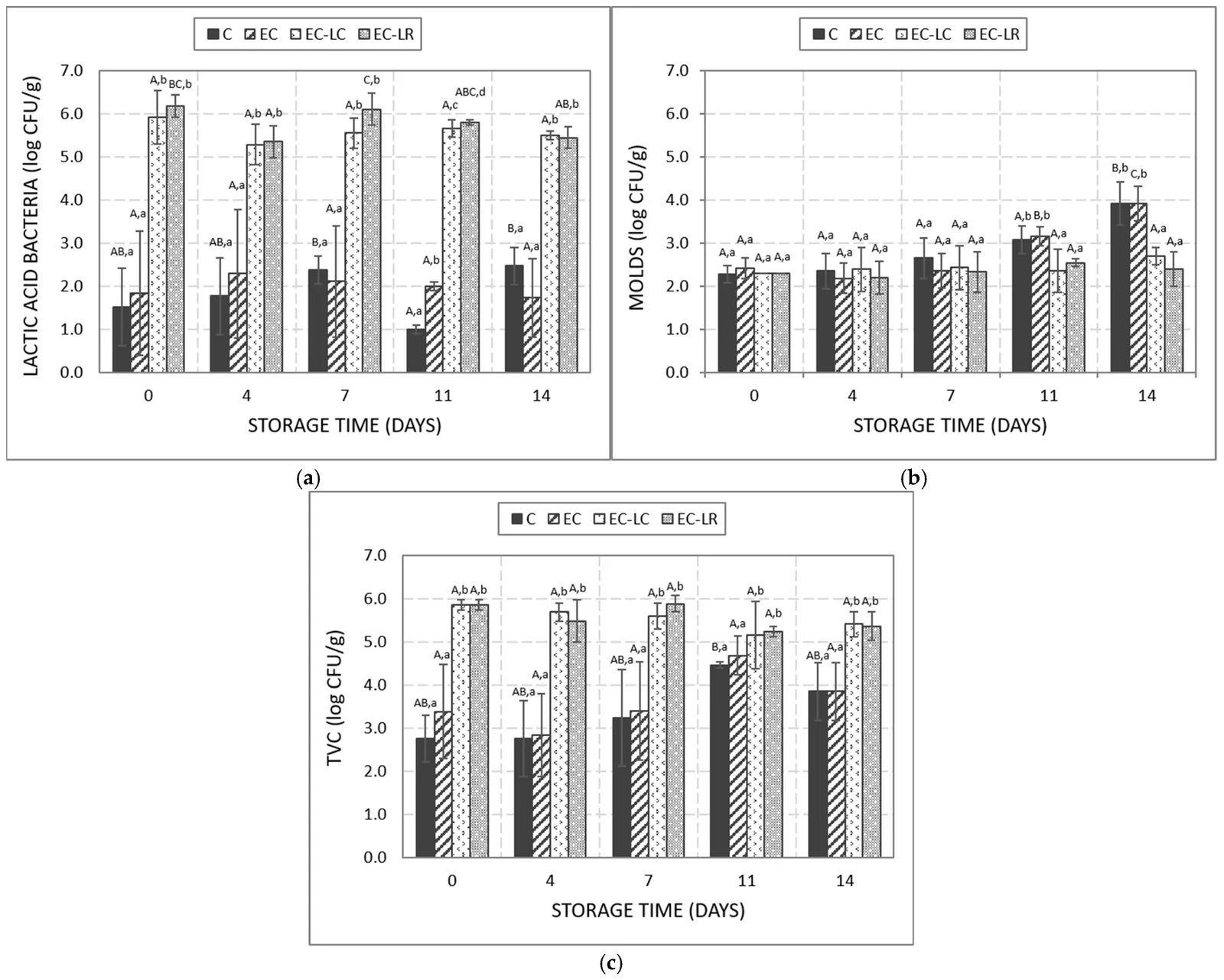
Variation in population (log CFU/g) of presumptive LAB (**a**), molds (**b**), and TVC (**c**) of strawberries coated with multilayer SA edible coatings supplemented with either *Lbc. casei* Shirota (EC-LC) or *Lbc. rhamnosus* GG (EC-LR) or no probiotic cultures (EC), packaged in food-contact macro-perforated PET containers and stored at 4 °C. Uncoated strawberries served as controls. Different uppercase letters within the same treatment indicate statistically significant differences among sampling days (*p* < 0.05). Different lowercase letters within the same sampling day indicate statistically significant differences among treatments (*p* < 0.05).

Figure 5 illustrates the variations in counts of presumptive LAB, molds, and TVC enumerated in strawberries over a 14-day storage period at 4 °C. The populations recovered from the controls and EC samples on MRS agar ranged from 1.0 to 2.5 log CFU/g (presumptive LAB) throughout storage (Figure 5a). On the other hand, the initial populations recovered from EC-LC and EC-LR samples on MRS agar were in the range of 5.9–6.2 log CFU/g, close to the targeted initial inoculum of probiotic cultures (Section 2.4), therefore confirming the successful incorporation of probiotic bacteria into the SA multilayer coating (Figure 5a). Moreover, the consistently high and relatively stable levels of *Lcb. casei* Shirota or *Lcb. rhamnosus* GG throughout storage at 4 °C, ranging from 5.5 to 6.2 log CFU/g, indicate that both studied probiotics survived within the biopolymer matrix and on the surface of strawberries (Figure 5a). Several previous studies have demonstrated the successful immobilization of probiotic cells in various combinations of coatings/films and strains, e.g., *Lb. rhamnosus* GG in rice starch-based, sodium caseinate, and high-viscosity SA [52,53], *L. acidophilus* and *L. rhamnosus* in CMC [54], and *L. plantarum* CECT 9567 together with a prebiotic (lactobionic acid) in delipidated egg-yolk proteins [55]. These studies reported that probiotic viability remained above 10^7^ CFU/g of film or coating during storage at 4 °C. Regarding molds, the presence of probiotic cultures (EC-LC and EC-LR samples) appeared to significantly inhibit fungal growth by 1.5–2.0 log CFU/g (*p* < 0.05) compared to the controls after 14 days of storage at 4 °C (Figure 5b). In contrast, the absence of probiotics (EC samples) allowed fungi to increase significantly (*p* < 0.05) during storage, reaching ca. 4.0 log CFU/g on day 14 (Figure 5b). Similar antifungal activity has been recorded in previous studies when *L. plantarum* or *L. rhamnosus* was incorporated in gelatin/inulin or CMC-coated strawberries, respectively, and stored at 4 °C for 16 days [26,29]. Moreover, researchers have reported the biocontrol activity of probiotic coatings against fungi such as *Bacillus cinerea* CECT 20518 and *Aspergillus niger* CECT 2805, when *L. plantarum* was incorporated into pregelatinized potato starch-coated or alginate-coated table grape berries during storage at 20 °C and 24 °C, respectively [56,57]. Competitive microbial interactions between LAB and fungi are usually related to several factors, e.g., competition for nutrients, production of metabolic substances (organic acids), and/or production of active compounds (i.e., peptides and VOCs) [25]. Moreover, it is well known that the breakdown of nutrients and fruit cell walls by the presence and growth of fungi may also contribute to post-harvest weight reduction [26,29,58], a result also observed in the present study (Figure 2a). Concerning TVC, the recorded populations in EC-LC and EC-LR samples (5.0–5.9 log CFU/g) (Figure 5c), which were comparable to the corresponding counts obtained on MRS agar (Figure 5a), suggest that the inoculated probiotics constituted a substantial proportion of the culturable microorganisms recovered in PCA from the probiotic-coated strawberries. Nevertheless, TVC may include various other microorganisms commonly found post-harvest in strawberries, namely yeasts, pseudomonads, and enterobacteria [59]. On the contrary, controls and EC samples showed a significant increase of ca. 1.5 log CFU/g (*p* < 0.05) during storage (Figure 5c). Overall, the presence of *Lcb. casei* Shirota or *Lcb. rhamnosus* GG at concentrations ≥ 10^6^ CFU/g (*p* ≥ 0.05) appeared to similarly suppress the proliferation of fungi and other indigenous spoilage microbiota, contributing to the microbiological shelf-life of the product.

### 3.7. Quantification of Organic Acids and Sugars via HPLC-UV/RI

The concentrations of the dominant organic acids (citric and malic acid) and sugars (glucose and fructose) were significantly affected by storage time and treatments (*p* < 0.05). Acetic, lactic, tartaric, succinic, and propionic acids were not detected during storage at 4 °C. Citric acid gradually decreased (*p* < 0.05) in controls from 1.16 ± 0.03 g/100 g (day 0) to 0.97 ± 0.05 g/100 g (day 14) (Table 2), thus reflecting the utilization of organic acids as substrates in respiration (tricarboxylic acid cycle) and senescence-related metabolism, as reported by previous researchers in various fruits [60,61]. A similar decreasing trend was observed for malic acid. Specifically, the concentration declined significantly in control samples during storage (*p* < 0.05), from 0.41 ± 0.06 g/100 g on day 0 to 0.32 ± 0.02 g/100 g on day 14 (Table 2). In contrast, the concentration of citric and malic acid (*p* ≥ 0.05) remained stable in multilayer SA-based coated samples with or without probiotics (EC, EC-LC, and EC-LR) throughout storage, indicating that edible coatings may slow down the metabolic activity of organic acid-degrading enzymes (e.g., malate dehydrogenase) by limiting oxygen availability, respiration rate, and organic acid catabolism [24,62]. Considering the absence of detectable lactic acid and changes in malic acid levels across all probiotic-coated strawberries (EC-LC and EC-LR) throughout storage, it can be concluded that neither lactic acid fermentation nor substantial malolactic fermentation occurred.

The concentrations of glucose and fructose were significantly (*p* < 0.05) increased during storage in all samples except for EC-LC samples (Table 2), while sucrose was not detected throughout storage in any sample. Specifically, in controls, glucose and fructose concentrations increased (*p* < 0.05) from 2.96 ± 0.10 to 3.94 ± 0.09 g/100 g and from 3.67 ± 0.07 to 4.63 ± 0.58 g/100 g, respectively, during storage. EC and EC-LR samples also showed increased glucose content (from 2.96 ± 0.10 to 3.63 ± 0.24 g/100 g and from 2.90 ± 0.03 to 3.06 ± 0.24 g/100 g, respectively) and fructose (from 3.39 ± 0.32 to 4.21 ± 0.56 g/100 g and from 3.45 ± 0.18 to 4.21 ± 0.12 g/100 g, respectively) throughout storage. It is well known that such an increase in sugars is potentially attributed to enzymatic hydrolysis of polysaccharides, cell wall degradation, and reduced firmness in untreated strawberries [63]. However, this interpretation appears particularly relevant for control samples, which at the same time exhibited the lowest recorded F_max_ values (*p* < 0.05) (Figure 4) and the highest population of pectinolytic microorganisms, such as fungi, throughout refrigerated storage (Figure 5b). In contrast, EC-LC samples showed increased firmness and significant inhibition of fungal growth (*p* < 0.05) throughout storage at 4 °C (Figure 4 and Figure 5b). Thus, the greater F_max_ observed in these samples may be associated with mechanisms such as alterations in carbohydrate and energy metabolism [64,65,66,67]. Similar results have recently been reported in SA-based coatings containing probiotics, i.e., *Lactococcus lactis*, *Lacticaseibacillus paracasei*, *Lcb. rhamnosus*, and *Lcb. plantarum*, which were associated with reduced sugar accumulation, delayed ripening, and improved overall post-harvest quality of strawberries [24,61]. Overall, EC-LC appeared to be the most effective in limiting changes in organic acid and sugar concentrations during refrigerated storage.

### 3.8. Sensory Evaluation

**Figure 6:**
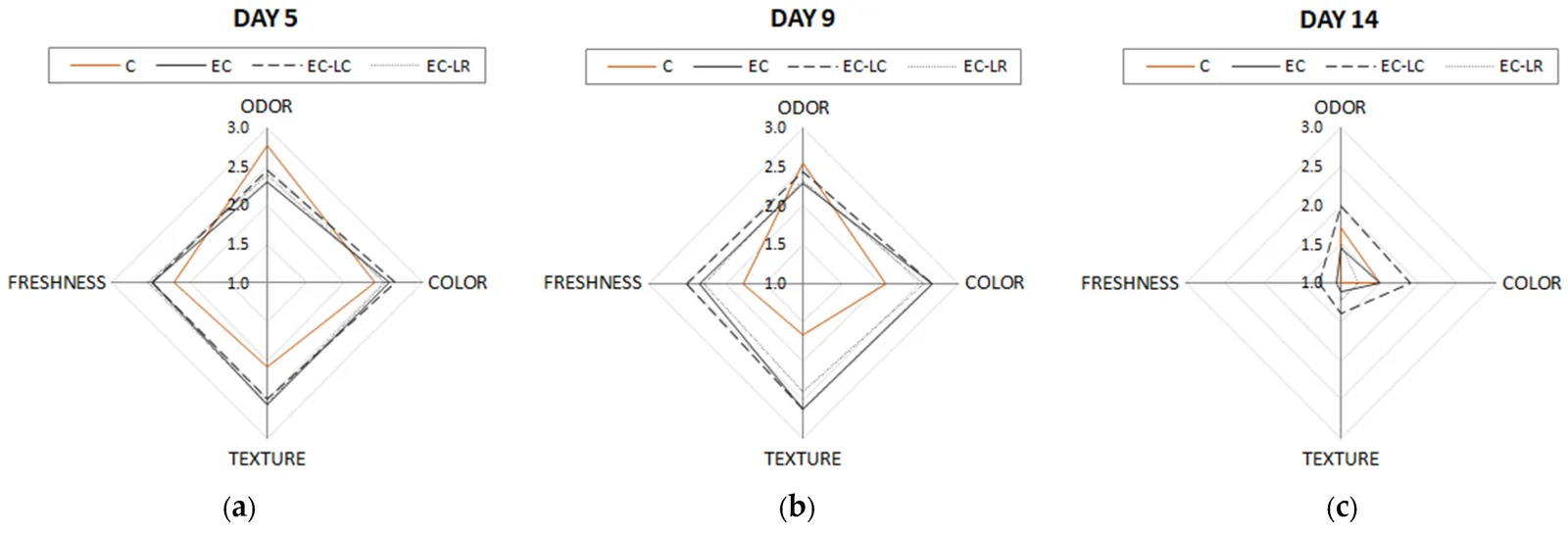
Sensory evaluation of odor, color, texture, and freshness of strawberries (mean values) coated with multilayer SA coating supplemented with either *Lbc. casei* Shirota (EC-LC) or *Lbc. rhamnosus* GG (EC-LR) or no probiotic cultures (EC), packaged in food-contact macro-perforated PET containers after (**a**) 5, (**b**) 9, and (**c**) 14 days of storage at 4 °C. Uncoated strawberries served as controls (C).

**Figure 7:**
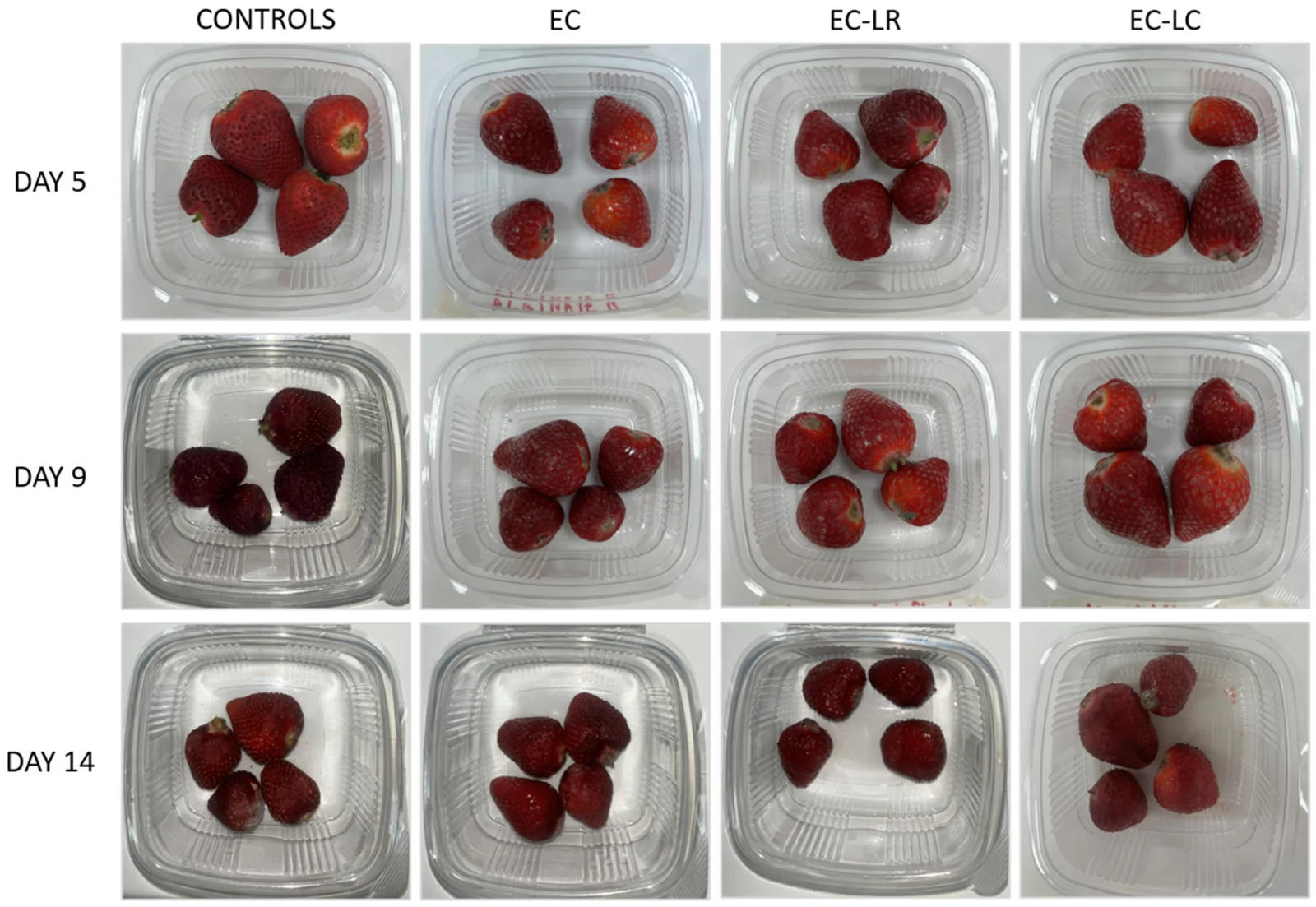
Representative photos of uncoated strawberries (controls) and multilayer SA-coated strawberries without probiotic cultures (EC) or multilayer SA-coated strawberries supplemented with *Lbc. casei* Shirota (EC-LC) or *Lbc. rhamnosus* GG (EC-LR), packaged in food-contact macro-perforated PET containers after 5, 9, and 14 days of storage at 4 °C.

Figure 6 demonstrates the variation in sensory attributes such as odor, color, texture, and freshness of multilayer probiotic-coated strawberries versus controls on days 5, 9, and 14 at 4 °C. The application of multilayer coating did not affect (*p* ≥ 0.05) the initial sensory parameters (day 0) in strawberries; namely, all treatments received excellent scores (from 2.5 to 3.0). On day 5, all coated strawberries received higher evaluation scores (ca. 2.5) than the controls across all sensory attributes except for odor, indicating a high level of acceptance by the panelists (Figure 6a). By day 9, the decline in scores of color, texture, and freshness of non-coated strawberries became more evident (*p* < 0.05), while the scores in odor remained unchanged compared to day 0 (*p* ≥ 0.05) (Figure 6b). The latter result is macroscopically visible in Figure 7, where the non-coated samples exhibited tissue shrinkage and surface darkening, as also recorded by firmness (Figure 4) and color measurements (Table 1), respectively. Previous studies have also reported high sensory acceptance of monolayer or multilayer coatings of various biopolymers (e.g., alginate/pectin, whey protein isolate/chitosan) compared to uncoated samples during refrigerated storage in fruits such as strawberries, fresh-cut pineapple, or watermelon [16,34,43]. In contrast, all coated samples (EC, EC-LC, and EC-LR) maintained high sensory quality, receiving scores ≥ 2.5 at all sensory attributes until day 9. This observation is also evident in Figure 7, which shows that the application of probiotic coatings preserved strawberry turgor, surface gloss, and vivid red color, consistent with the instrumental measurements of texture (Figure 4) and color (Table 1). No significant differences (*p* ≥ 0.05) were observed between the two probiotic cultures studied until the ninth day of storage. On day 14, all samples became unacceptable except for EC-LC samples, which remained marginally acceptable to the consumers in terms of odor and color scores (Figure 6c). Previous studies have reported that SA active packaging of various fruits such as fresh blueberries or minimally processed apples with *Lcb. rhamnosus* and/or *Bifidobacterium animalis* subsp. *lactis* enhanced freshness and overall acceptability for up to 10–14 days under refrigerated storage [50,68,69].

## 4. Conclusions

The application of the LBL coating technique incorporating *Lcb. casei* Shirota or *Lcb. rhamnosus* GG showed clear potential to enhance the post-harvest quality and shelf-life of strawberries during storage at 4 °C. Based on the results, both probiotic-enriched SA coatings inhibited weight loss and TSS accumulation, thereby delaying strawberry ripening. Concerning the texture, the application of *Lcb. casei* Shirota (EC-LC) multilayer SA coating on strawberries enhanced their textural integrity compared to *Lcb. rhamnosus* GG (EC-LR). All coated samples showed a smaller decline in the a* parameter (redness) than controls, while EC-LC reached the highest values of lightness during storage, indicating a glossier appearance of the product. Moreover, fungal growth was inhibited, while the two embedded probiotic strains remained viable, with counts near 10^6^ CFU/g of fruit during the 14-day storage at 4 °C. Based on these values, consumption of a minimum of 100 g of strawberries per day would deliver an estimated functional dose of 10^8^ CFU, which falls within the typical range of commonly reported daily probiotic intakes (10^8^–10^11^ CFU/day) [36,70]. Finally, strawberries with probiotic coatings received high sensory scores and consumer acceptability compared to controls after 9 days of storage at 4 °C, thereby extending their conventional marketable shelf-life. Notably, strawberries coated with *Lcb. casei* Shirota (EC-LC) maintained marginal consumer acceptability in terms of odor and color attributes until the 14th day of storage. To enhance the applicability of such systems, future research should include: (i) dynamic storage scenarios to better assess the practical applicability of such active packaging applications across the cold chain, (ii) in vitro digestion studies to evaluate the survival and release of probiotics throughout the gastrointestinal tract, and (iii) clinical studies to confirm the functional food potential of probiotic-coated strawberries in humans, including probiotic viability after consumption and potential health effects.

## Figures and Tables

**Table 1:** Color variation (L*, a*, b* parameters) of strawberries coated with multilayer SA edible coatings supplemented with either *Lbc. casei* Shirota (EC-LC) or *Lbc. rhamnosus* GG (EC-LR) or no probiotic cultures (EC), packaged in food-contact macro-perforated PET containers and stored at 4 °C. Uncoated strawberries served as controls.

		Storage Days				
	Treatment	0	4	7	11	14
L* parameter	CONTROL	35.77 ± 0.13 ^A,a^	36.03 ± 2.71 ^A,a^	35.65 ± 0.73 ^A,a^	35.18 ± 1.56 ^A,a^	36.18 ± 0.16 ^A,a^
	EC	35.73 ± 0.05 ^A,a^	39.72 ± 0.41 ^C,c^	40.37 ± 2.28 ^C,b^	38.05 ± 0.69 ^B,b^	38.80 ± 1.62 ^BC,b^
	EC-LC	35.73 ± 0.05 ^A,a^	38.94 ± 0.28 ^B,bc^	41.57 ± 2.30 ^C,b^	39.00 ± 2.16 ^B,b^	39.59 ± 2.38 ^B,b^
	EC-LR	35.73 ± 0.05 ^A,a^	38.11 ± 0.57 ^BC,b^	40.28 ± 0.59 ^D,b^	37.76 ± 0.44 ^B,b^	39.27 ± 0.34 ^CD,b^
a* parameter	CONTROL	35.91 ± 0.03 ^C,b^	33.84 ± 1.48 ^B,b^	32.83 ± 0.47 ^B,c^	31.54 ± 1.04 ^A,b^	30.83 ± 0.50 ^AB,b^
	EC	31.02 ± 0.08 ^C,a^	30.03 ± 0.43 ^BC,a^	29.56 ± 0.46 ^B,b^	29.33 ± 1.48 ^A,a^	28.72 ± 1.06 ^AB,a^
	EC-LC	31.02 ± 0.08 ^B,a^	30.60 ± 0.99 ^B,a^	28.89 ± 0.53 ^A,a^	27.94 ± 1.12 ^A,a^	28.29 ± 1.21 ^A,a^
	EC-LR	31.02 ± 0.08 ^C,a^	29.46 ± 0.72 ^BC,a^	27.86 ± 1.06 ^A,a^	28.37 ± 0.26 ^A,a^	29.35 ± 0.04 ^B,a^
b* parameter	CONTROL	23.02 ± 0.42 ^B,b^	21.00 ± 2.00 ^A,b^	20.04 ± 2.27 ^A,b^	20.44 ± 1.89 ^A,b^	21.98 ± 0.21 ^AB,b^
	EC	18.19 ± 0.02 ^A,a^	19.85 ± 1.61 ^C,b^	19.09 ± 0.12 ^BC,b^	18.57 ± 0.40 ^BC,ab^	17.78 ± 1.07 ^AB,a^
	EC-LC	18.19 ± 0.02 ^A,a^	19.64 ± 0.21 ^B,b^	18.36 ± 1.18 ^B,ab^	20.48 ± 0.96 ^B,b^	18.53 ± 2.04 ^B,a^
	EC-LR	18.19 ± 0.02 ^A,a^	17.13 ± 1.79 ^AB,a^	17.23 ± 1.64 ^AB,a^	18.67 ± 0.61 ^B,a^	18.86 ± 0.94 ^B,a^

Different uppercase letters within the same treatment indicate statistically significant differences among sampling days (*p* < 0.05). Different lowercase letters within the same sampling day indicate statistically significant differences among treatments (*p* < 0.05).

**Table 2:** Variation in organic acids (citric and malic acid) (g/100 g) and sugars (glucose and fructose) (g/100 g) of strawberries coated with multilayer SA edible coatings supplemented with either *Lbc. casei* Shirota (EC-LC) or *Lbc. rhamnosus* GG (EC-LR) or no probiotic cultures (EC), packaged in food-contact macro-perforated PET containers and stored at 4 °C. Uncoated strawberries served as controls.

Concentration (g/100 g)		Storage Time (Days)		
	Treatments	0	7	14
CITRIC ACID	CONTROL	1.16 ± 0.03 ^B,b^	1.09 ± 0.15 ^AB,b^	0.97 ± 0.05 ^A,b^
	EC	0.95 ± 0.10 ^A,a^	0.83 ± 0.07 ^A,a^	0.94 ± 0.01 ^A,b^
	EC-LC	0.95 ± 0.16 ^A,a^	0.78 ± 0.08 ^A,a^	0.81 ± 0.07 ^A,a^
	EC-LR	0.98 ± 0.02 ^A,ab^	0.86 ± 0.12 ^A,a^	0.90 ± 0.04 ^A,b^
MALIC ACID	CONTROL	0.41 ± 0.06 ^B,a^	0.34 ± 0.02 ^A,b^	0.32 ± 0.02 ^A,b^
	EC	0.33 ± 0.06 ^A,a^	0.29 ± 0.01 ^A,ab^	0.30 ± 0.03 ^A,b^
	EC-LC	0.33 ± 0.03 ^A,a^	0.27 ± 0.02 ^A,a^	0.26 ± 0.03 ^A,a^
	EC-LR	0.36 ± 0.03 ^A,a^	0.32 ± 0.04 ^A,ab^	0.31 ± 0.01 ^A,b^
GLUCOSE	CONTROL	2.96 ± 0.10 ^A,a^	3.18 ± 0.05 ^B,b^	3.94 ± 0.09 ^C,c^
	EC	2.96 ± 0.10 ^A,a^	2.56 ± 0.29 ^A,a^	3.63 ± 0.24 ^B,b^
	EC-LC	2.75 ± 0.14 ^A,a^	2.36 ± 0.32 ^A,a^	2.33 ± 0.31 ^A,a^
	EC-LR	2.90 ± 0.03 ^A,a^	2.70 ± 0.26 ^A,a^	3.06 ± 0.24 ^Β,a^
FRUCTOSE	CONTROL	3.67 ± 0.07 ^A,a^	4.09 ± 0.84 ^A,a^	4.63 ± 0.58 ^A,b^
	EC	3.39 ± 0.32 ^A,a^	3.30 ± 0.22 ^A,a^	4.21 ± 0.56 ^B,ab^
	EC-LC	3.54 ± 0.08 ^A,a^	3.16 ± 0.36 ^A,a^	3.39 ± 0.44 ^A,a^
	EC-LR	3.45 ± 0.18 ^A,a^	3.54 ± 0.29 ^A,a^	4.21 ± 0.17 ^B,ab^

Different uppercase letters within the same treatment indicate statistically significant differences among sampling days (*p* < 0.05). Different lowercase letters within the same sampling day indicate statistically significant differences among treatments (*p* < 0.05).

## Data Availability

The raw data supporting the conclusions of this article will be made available by the authors on request.
